# Délégation de tâches de vaccination aux acteurs communautaires: stratégie innovante pour atteindre la cible vaccinale lors d’une campagne de vaccination contre la rougeole-rubéole dans une zone à fort défi sécuritaire, Ouargaye, Burkina Faso, 2024

**DOI:** 10.11604/pamj.2025.50.89.45931

**Published:** 2025-04-01

**Authors:** Morou Nikiema, Mahamoudou Nyantudre, Ange Anouck Désiré Kabore, Patrice Komboigo, Amidou Gnakini, Dénis Yelbeogo, Ousmane N°2 Kalmogo, Roland Wendinpassédé Nouktara, Saidou-Mady Bagaya, Issa Guire

**Affiliations:** 1 District Sanitaire de Ouargaye, Région du Centre Est, Ouargaye, Burkina Faso,; 2 Coordination de la Formation en Epidémiologie de Terrain, Ouagadougou, Burkina Faso,; 3 District Sanitaire de Pouytenga, Région du Centre Est, Pouytenga, Burkina Faso,; 4 Direction Régionale de la Santé du Centre Est, Ouagadougou, Burkina Faso,; 5 Ministère de la Santé, Ouagadougou, Burkina Faso

**Keywords:** Vaccination, communautaire, rougeole, Burkina Faso, Vaccination, community, measles, Burkina Faso

## Abstract

**Introduction:**

les conflits impactent négativement les indicateurs de vaccination. Pour améliorer les couvertures vaccinales, le district sanitaire de Ouargaye à défi sécuritaire a formé des acteurs communautaires de certaines formations sanitaires fermées sur la vaccination. La présente étude vise à mettre en évidence l'apport de ces acteurs communautaires, à l'atteinte de la couverture vaccinale lors de la campagne de vaccination contre la rougeole-rubéole du 15 au 21 mars 2024.

**Méthodes:**

il s'est agi d'une étude transversale allant du 1^er^ mars au 30 avril 2024. L'échantillon était composé de tous les acteurs communautaires formés sur la vaccination et toute la cible de la campagne de vaccination contre la rougeole-rubéole issus des neuf formations sanitaires concernées par la stratégie. Les données ont été collectées grâce à un canevas d'analyse documentaire. Nous avons évalué la contribution des acteurs communautaires à l'atteinte de la couverture vaccinale à l'aide d'un test de Chi 2. Le seuil de significativité était de 5%.

**Résultats:**

à l'issue des sept jours de campagne, 5403 enfants ont été vaccinés par les acteurs communautaires, soit 28,65% (5403/18853) de la cible. Les acteurs communautaires ont contribué à l'atteinte de la couverture vaccinale des neuf formations sanitaires concernées. Cette contribution était statistiquement significative (p-value < 2.2e-16). Sur les 5 403 enfants vaccinés par les acteurs communautaires, seulement quatre cas de manifestations indésirables post-vaccinales ont été notifiés.

**Conclusion:**

la délégation des tâches de vaccination aux acteurs communautaires dans les zones à défis sécuritaire permet d'améliorer les couvertures vaccinales lors des campagnes de vaccination.

## Abstract (en)

**Introduction:**

conflicts negatively impact vaccination indicators. To improve vaccination coverage, the Ouargaye health district an area facing security challenges trained community actors from certain closed health facilities on vaccination. This study aims to highlight the contribution of these community actors to achieving vaccination coverage during the measles-rubella vaccination campaign held from March 15 to 21, 2024.

**Methods:**

a cross-sectional study was conducted from March 1 to April 30, 2024. The sample included all community actors trained in vaccination and the entire target population of the measles-rubella vaccination campaign across the nine health facilities concerned by the strategy. Data were collected using a documentary analysis framework. The contribution of community actors to vaccination coverage was assessed using a Chi-square test, with a significance threshold set at 5%.

**Results:**

over the seven days of the campaign, 5,403 children were vaccinated by community actors, representing 28.65% (5,403/18,853) of the target population. Community actors contributed significantly to achieving vaccination coverage in the nine targeted health facilities. This contribution was statistically significant (p-value < 2.2e-16). Among the 5,403 children vaccinated by community actors, only four cases of adverse post-vaccination events were reported.

**Conclusion:**

Delegating vaccination tasks to community actors in high-security risk areas enhances vaccination coverage during immunization campaigns.

## Introduction

La vaccination est un succès en termes de santé et de développement dans le monde et permet de sauver des millions de vies chaque année [1,2].

Cependant, le succès des différents programmes de vaccination reste insuffisant dans certains pays surtout dans les pays en proie aux conflits armés [3-8]. A travers le monde, environ 40% des enfants non vaccinés ou insuffisamment vaccinés vivent dans des pays qui sont partiellement ou entièrement touchés par des conflits [8]. Ces faibles couvertures vaccinales dans ces localités s'expliquent par l'impact des crises sur le système de santé avec perturbation de l'offre des services de vaccination [9]. Cette situation expose fortement les populations de ces localités augmentant ainsi le risque d'émergence et/ou de réémergence de certaines pathologies évitables par la vaccination. Il revient donc à chaque gouvernement de mettre en place des approches innovantes pour assurer une bonne couverture vaccinale dans ces zones à risques.

Le Burkina Faso, un pays touché par une crise sécuritaire depuis 2015, s'est engagé dans une dynamique d'amélioration de la préparation et la réponse aux situations d'urgences sanitaires. C'est ainsi que le Ministère de la santé et de l'hygiène publique (MSHP) à travers la direction de la prévention par les vaccinations (DPV) a élaboré et mis à la disposition des acteurs des régions à défis sécuritaires, une stratégie nationale de vaccination dans les zones à défi sécuritaire (ZADS) [10]. Dans ladite stratégie, certaines tâches qui étaient autrefois dévolues aux agents de santé ont été déléguées aux acteurs communautaires que sont les agents de santé à base communautaire (ASBC) issus des zones où les formations sanitaires sont fermées ou fonctionnent à minima du fait de la crise sécuritaire. Lesdits agents de santé à base communautaire (ASBC) ont été recrutés sur le plan national en 2016, en raison de deux ASBC par village administratif. Ils avaient en charge la mise en œuvre des activités promotionnelles dans leurs villages respectifs.

En s'inspirant de la stratégie nationale de vaccination dans les zones à défi sécuritaire, le district sanitaire de Ouargaye, un des districts touchés par l'insécurité a bénéficié en 2023 de l'accompagnement financier de l'USAID à travers le projet *Momentum Integrated Health Resilience (MIHR)* pour la formation de vingt-sept (27) ASBC issus de l'aire sanitaire de neuf (9) formations sanitaires fermées ou fonctionnant à minima sur cette délégation de tâches de vaccination en vue de sa mise en œuvre au niveau du district. Ces agents de santé à base communautaire ont bénéficié d'une formation de douze (12) jours sur la vaccination, comprenant trois jours de phase théorique, et neuf (9) jours de phase pratique dont trois (3) sur modèle anatomique et six (6) dans les formations sanitaires fonctionnelles voisines. En plus, des activités promotionnelles relevant de leur paquet d'activité habituel, ces agents de santé à base communautaire appuient le district dans la conduite de la vaccination de routine et lors des campagnes de vaccination. Avant la mise en œuvre de cette stratégie de délégation des tâches de vaccination aux ASBC, les couvertures vaccinales administratives en rougeole-rubéole 2^e^ dose variaient entre 41,4 à 88,5% dans les neuf formations sanitaires concernées. Ces dites couvertures étaient au-dessus de 95% dans toutes les neuf formations sanitaires avant l'éclosion de la crise sécuritaire. De façon générale, la présente étude vise à décrire l'apport de ces vingt-et-sept (27) agents de santé à base communautaire, formés sur la vaccination à l'atteinte de la cible vaccinale du district lors de la mise en œuvre de la campagne de vaccination contre la rougeole-rubéole qui s'est déroulée du 15 au 21 mars 2024.

De façon spécifique il s'est agi de: décrire l'organisation de la campagne de vaccination contre la rougeole-rubéole au niveau communautaire par les ASBC; estimer l'apport des ASBC à l'atteinte de la cible vaccinale lors de la campagne de vaccination contre la rougeole rubéole; identifier les principales difficultés ayant entravé le bon déroulement de la campagne de vaccination.

## Méthodes

**Type et période d'étude:** il s'est agi d'une étude transversale qui s'est déroulée du 1^er^ mars au 30 avril 2024.

**Cadre de l'étude:** notre étude s'est déroulée au district sanitaire de Ouargaye dans la province du Koulpélogo, région du Centre-Est. Ce district couvre une superficie de 5656 Km^2^ et comporte 42 formations sanitaires dont 2 centres médicaux (CM). Au moment de l'étude, du fait de la crise sécuritaire, on dénombrait 16 formations sanitaires fermées et douze autres fonctionnant à minima. Les formations sanitaires fonctionnant à minima, sont celles qui n'arrivaient plus à offrir la totalité de leur paquet de soins dans le temps et dans l'espace pour cause d'insécurité, dont la suspension des stratégies avancées de vaccination [11]. Pour la première phase de la mise en œuvre de la délégation des tâches de vaccination aux acteurs communautaires, neuf (9) des vingt-et-huit (28) formations sanitaires fermées et fonctionnant à minima ont été retenues sur la base des couvertures vaccinales. Notre étude s'est déroulée sur les aires sanitaires de ces neuf (9) formations sanitaires dont 2 étaient fermées (Zoaga et Bittin) et 7 fonctionnant à minima (Kaongho, Salembaoré, Zembendé, Comin-Yanga, Kobré, Kohogo et Bousgou). Certains villages de ces formations sanitaires abritent des personnes déplacées internes.

**Population d'étude et échantillonnage:** ont été concernés par cette étude ; l'ensemble des vingt-et-sept (27) ASBC issus des zones touchées par l'insécurité et ayant bénéficié de la formation sur la vaccination ; les enfants cibles de la campagne de vaccination contre la rougeole-rubéole. Ce sont les enfants de 9-59 mois, estimés à 18 853 pour les neuf (9) formations sanitaires concernées par l'étude. L'échantillonnage était donc de type exhaustif.

**Collecte des données:** la principale technique utilisée pour la collecte des données était l'analyse documentaire. Grâce à un canevas d'analyse des données, les fiches synthèses journalières des données de la campagne rougeole-rubéole des formations sanitaires concernées, les masques de saisie des données de la campagne des formations sanitaires concernées et les rapports de formation des ASBC sur la vaccination ont constitué nos sources d'information. Les principales variables qui ont été collectées sont: âge des ASBC, sexe des ASBC, niveau d'études des ASBC, résidence des ASBC, année de recrutement des ASBC, activités antérieures des ASBC, âge des enfants cibles, statut de scolarisation, préparation des équipes de vaccination, constitution des équipes, ravitaillement en vaccins et consommables, sites de vaccinations, répartition des tâches, suivi et supervision, rendu des résultats, précautions sécuritaires, nombre d'enfants vaccinés, cible, nombre de manifestations post-vaccinales indésirables (MAPI) notifiées, les difficultés rencontrées.

**Analyse des données:** les données collectées ont été saisies, traitées et analysées sur un ordinateur muni de Microsoft office 2010. Nous avons par la suite calculé la moyenne d'âge des ASBC et les proportions d'enfants vaccinés par les ASBC à l'aide d’Excel 2010. Nous avons évalué la contribution des ASBC à l'atteinte de la couverture vaccinale à l'aide d'un test de Chi 2. Le seuil de significativité était de 5%. Nous avons comparé les couvertures vaccinales atteintes sans la participation des ASBC et avec leur participation.

**Aspects éthiques et déontologiques:** les autorités administratives ont jugé qu'une clairance éthique n'était pas nécessaire et ont donné une autorisation administrative avant la collecte des données. Les données collectées ont été traité dans l'anonymat et en toute confidentialité.

## Résultats

**Caractéristiques socio-démographiques des ASBC formés sur la vaccination et des enfants vaccinés**

Au total, vingt-et-sept (27) agents de santé à base communautaire (ASBC) ont bénéficié de la formation sur la vaccination dans les neuf formations sanitaires. Leur âge moyen était de 38,92 ans avec des extrêmes allant de 25 ans à 55 ans. Le sexe ratio était de 8 hommes pour une femme. Tous les vingt-et-sept (27) ASBC ont le niveau d'étude primaire. Ils savent lire et écrire. C'était une des principales conditions pour les enrôler dans la formation sur la délégation des tâches de vaccination. Ils résident tous dans leurs villages d'origines. Tout comme la population des villages concernés, les ASBC pratiquent des activités agro-pastorales en plus des activités de santé communautaires promotionnelles qu'ils mènent régulièrement. Ils ont été recrutés en 2016. Le Tableau 1 fait la répartition du nombre d'ASBC formés sur la vaccination par formation sanitaire. Pour ce qui concerne les enfants vaccinés par les ASBC, 92,05% (4974/5403) de ces enfants étaient âgés de 12 à 59 mois et 7,95% appartenaient à la tranche d'âge de 9-11 mois. Aucun de ces enfants ne partaient à l'école au moment de l'étude du fait de la fermeture des établissements scolaires dans les villages concernés à cause de la situation sécuritaire.

**Tableau 1:** situation des enfants vaccinés contre la rougeole-rubéole par les ASBC formés sur la vaccination par formations sanitaires lors de la campagne de vaccination du 15 et le 21 mars 2024, district sanitaire de Ouargaye, région du Centre Est, Burkina Faso

Formations sanitaires	Nombre d'ASBC formés	Enfants cibles 9-59mois	Enfants 9-59 mois vaccinés par les ASBC	Proportion des enfants vaccinés par les ASBC (%)
Bittin	2	1398	390	27,9
Bousgou	3	978	108	11,04
Comin-Yanga	4	4012	1611	40,15
Kaongho	3	2858	493	17,25
Kobré	3	578	30	5,19
Kohogo	4	2474	389	15,72
Salembaoré	3	1215	1045	86
Zembendé	3	3750	1150	30,67
Zoaga	2	1590	187	11,76
**Total formations sanitaires**	**27**	**18853**	**5403**	**28,65**
**Total district**	**27**	**64057**	**5403**	**8,43**

ASBC= agent de santé à base communautaire

**Organisation d'un poste de vaccination au niveau communautaire par les ASBC**

***Préparation des équipes de la campagne de vaccination contre la rougeole:*** les ASBC formés sur la vaccination ont bénéficié, d'un briefing d'un jour sur la campagne de vaccination contre la rougeole-rubéole avec les responsables des formations sanitaires fonctionnelles voisines. Les objectifs de la campagne, les cibles, les techniques de dilution et d'administration du vaccin ont été présentés lors de cette rencontre. Lors de cette activité, une cartographie des sites de vaccination a été réalisée. Au regard des difficultés de déplacement et du nombre de dose dans un flacon du vaccin, la stratégie porte à porte a été peu recommandée.

***Constitution des équipes de la campagne de vaccination contre la rougeole:*** une équipe est constituée de deux agents de santé à base communautaires dont au moins un formé sur la délégation des tâches. Une troisième personne qui est dans la plupart des cas, un membre influent de la communauté est chargée de la mobilisation. Ce dernier, passe de porte en porte pour faire sortir la cible tout en évitant de mobiliser beaucoup de personnes.

***Site de vaccination:*** le nombre de sites de vaccination était variable selon la superficie des villages ; deux, trois, voire quatre sites par village ont été mis en place. Chaque village a été subdivisé en quartier et parfois en sous quartier pour éviter les grands rassemblements. Chaque quartier ou sous quartier a constitué un site de vaccination. Dans d'autres villages, les grandes concessions ont abrité des sites de vaccination.

***Répartition des tâches sur le terrain:*** l'ASBC formé sur la délégation des tâches, réalise la dilution et l'administration du produit vaccinal tandis que le deuxième organise les enfants, assure la communication interpersonnelle, renseigne la documentation notamment la fiche de coche, les carnets et cartes de vaccination des enfants. Il les invite à rentrer chez eux après avoir reçu leurs doses, pour éviter les attroupements. Le troisième volontaire est chargé de la mobilisation.

***Suivi et supervision:*** le suivi de la mise en œuvre de la campagne a été fait pour la plupart des équipes au téléphone, mais dans certains cas, à travers des informations collectées auprès des communautés.

***Rendu quotidien des résultats:*** durant la campagne de vaccination, un débriefing quotidien entre les ASBC et les responsables des formations sanitaires est réalisé. Les fiches de coches et les stocks restants sont vérifiés pour s'assurer du bon remplissage des outils et de la concordance des chiffres entre stocks reçus, utilisés et restants. Les difficultés rencontrées sur le terrain sont également passées au peigne fin et des solutions sont proposées pour un bon déroulement de la campagne.

***Précautions sécuritaires:*** au cours de la formation, un accent particulier a été mis sur la conduite pratique de l'activité sur le terrain. Les grands rassemblements sont fortement déconseillés. Les ASBC doivent constituer de petits groupes. Les forces de défenses et de sécurité ainsi que les volontaires pour la défense de la patrie ont été mis en contribution. Ils sont informés des sites et du programme de vaccination des différentes équipes. Pour plus de précautions, les ASBC concernés ont porté des dossards (de la campagne polio) qui permettaient de les identifier systématiquement.

**Nombre d'enfants vaccinés contre la rougeole par les ASBC formés sur la vaccination**

Au total, dans ces neuf formations sanitaires concernées par l'étude, 18 853 enfants de 9-59 mois constituaient la cible de la campagne de vaccination contre la rougeole-rubéole.

A l'issue des sept (7) jours de campagne, 5 403 enfants ont été vaccinés par les vingt-et-sept (27) ASBC formés sur la vaccination, soit 28,65% (5 403/18 853) de la cible des neuf (9) formations sanitaires concernées. En moyenne, chaque ASBC a vacciné environ 29 enfants par jour durant les sept (7) jours de la campagne. Autour de 1% de ces enfants n'avaient reçu aucune dose du vaccin rougeole-rubéole auparavant. Les ASBC ont contribué à l'atteinte de la couverture vaccinale des neuf (9) CSPS concernés et cette contribution était statistiquement significative (p-value < 2.2e-16). Selon notre test statistique, si les agents de santé avaient travaillé seuls, la couverture vaccinale aurait été inférieure d'environ 26-27% (IC=[-0.2700642, -0.2579850]). A l'échelle du district, 8,43% (5 403/64 057) de la cible ont été vaccinés par les ASBC. Le Tableau 1 fait la synthèse des enfants vaccinés par les ASBC lors de la campagne de vaccination contre la rougeole-rubéole.

***Notification des manifestations indésirables post vaccinales (MAPI):*** sur les 5403 enfants vaccinés par les ASBC, 4 cas de MAPI ont été notifiées par les ASBC de la formation sanitaire de Kohogo, dont un cas de fébricule et trois cas d'œdème au site de vaccination.

**Les difficultés ayant entravé le bon déroulement de la campagne**

Les principales difficultés rencontrées par les ASBC portaient sur les difficultés de ravitaillement dû à la réduction de la mobilité, l'absence de moyens de déplacements, les difficultés d'acquisition des Ice box localement, le nombre très insuffisant d'ASBC formés sur la délégation des tâches, l'absence de réseau téléphonique dans certaines localités pour faciliter les comptes rendus aux agents des CSPS proches.

## Discussion

**De l'organisation des sites de la campagne de vaccination par les ASBC :** l'organisation de la campagne de vaccination par les ASBC en communauté s'aligne sur les directives de la direction de la prévention par les vaccinations en matière d'organisation des sites de vaccination. En effet chaque équipe comprend trois personnes comme le recommande les directives. La campagne se déroulant en zone à défi sécuritaire, les ASBC ont dû adopter des initiatives locales, telle la collaboration étroite avec les forces de défenses et de sécurité, le port de signes distinctifs tels que les dossards, les portes-vaccins, l'implication des communautés elles-mêmes, la constitution de petits groupes pour la vaccination. Aussi, l'appartenance des ASBC à la communauté crée un sentiment de confiance entre eux et toute la population. Ils ont donc accès et connaissent pratiquement tous les enfants cibles de la campagne. Cette approche est partagée par Nnadi *et al*. au Nigeria qui dans leur étude proposaient l'utilisation des équipes locales de vaccination comme une approche pour toucher la cible dans les zones à conflit [4].

**Du nombre d'enfants vaccinés contre la rougeole-rubéole par les ASBC formés sur la vaccination:** durant les sept (7) jours de campagne (du 15 au 21 mars 2024), chaque ASBC a vacciné en moyenne, 29 enfants par jour. Ce chiffre est bien inférieur aux prévisions nationales selon lesquelles, chaque équipe vaccinale devrait vacciner par jour autour de 50 enfants dans les zones à défis sécuritaires. Cette situation est liée à la cible; les ASBC devant vacciné uniquement les enfants de leur village d'origine afin de limiter les déplacements; et aux difficultés de ravitaillement. Environ 28,65% des enfants cibles des neuf formations sanitaires ont été vaccinés par les ASBC. Ce résultat fort encourageant, montre une fois de plus que dans les zones conflictuelles, la délégation des tâches aux acteurs communautaires constitue une alternative. Peu de MAPI ont été notifiées. Ce qui pourrait s'expliquer par la bonne maitrise de la technique vaccinale par les ASBC. Notons qu'ils ont été formés depuis septembre 2023 et font régulièrement des activités de vaccination de routine dans leurs villages respectifs. Nonobstant ce fait, les 4 MAPI rapportées étaient toutes mineures (fièvre, et gonflements aux points d'injection).

**Des difficultés rencontrées :** les principales difficultés vécues par ces ASBC sont en lien avec le ravitaillement en vaccins, la difficulté pour rallier certains quartiers éloignés, l'absence de chaine de froid à proximité. La construction et l'équipement de postes de santé communautaire dans certaines localités permettraient d'amoindrir cette difficulté. Dans une revue systématique, Lam *et al*. ont relevé l'insécurité croissante, la destruction de la chaine de froid et des infrastructures comme principaux défis à la mise en œuvre des stratégies de vaccination dans les zones conflictuelles [12].

## Conclusion

La campagne de vaccination contre la rougeole-rubéole s'est déroulée sans incidents dans l'ensemble des neuf (9) formations sanitaires bénéficiaires de cette approche innovante. Plus du quart de la cible de ces formations sanitaires ont été vaccinés par les ASBC. La délégation de tâches aux acteurs locaux est une alternative efficace à l'atteinte des couvertures vaccinales lors des activités de vaccination supplémentaire dans les zones à fort défi sécuritaire. La mise en œuvre d'approches visant à approvisionner régulièrement ces équipes locales permettrait de rehausser encore la couverture vaccinale et d'assurer une vaccination de qualité.

### Etat des connaissances sur le sujet

- Les situations de crise impactent négativement les programmes de vaccination car elles entrainent une dislocation de l'appareil sanitaire ;
- Peu d'initiatives communautaires sont développées pour rehausser les couvertures vaccinales dans ces zones ;
- Les quelques initiatives développées ne sont pas documentées.

### Contribution de notre étude à la connaissance

- L'identification d'acteurs communautaires suivi de leur capacitation sur la vaccination a contribué de façon significative à l'augmentation des couvertures vaccinales lors de la campagne de vaccination contre la rougeole-rubéole dans un contexte de défi sécuritaire, réduisant ainsi le risque d'occurrence épidémique dans ces zones;
- Les communautés peuvent pleinement participer à leur santé dans les zones à défis sécuritaire.

## Remerciements

Nos remerciements à toute l'équipe de la DRS du Centre-Est pour l'appui technique ayant permis d'opérationnaliser la délégation des tâches de vaccination aux ASBC. Toute notre gratitude à l'équipe du projet MIHR/USAID pour l'appui financier ayant permis de conduire à terme le processus de délégation des tâches de vaccination aux ASBC. Merci pour l'accompagnement dont les ASBC formés sur la vaccination ont bénéficié. Merci pour aux ASBC pour votre résilience et votre engagement pour la santé de la population surtout la frange infantile.
